# Supplementary material for: Non-Achievement of Alanine Aminotransferase Normalization Associated with the Risk of Hepatocellular Carcinoma during Nucleos(t)ide Analogue Therapies: A Multicenter Retrospective Study
Source: J Clin Med. 2022 Apr 22;11(9):2354. doi: 10.3390/jcm11092354 (PMC9101732; doi:10.3390/jcm11092354)
Supplement: Supplementary file 1 [file jcm-11-02354-s001.zip › jcm-1656083-SI.pdf]

Non-Achievement of Alanine Aminotransferase Normalization is Associated with the Risk of Hepatocellular Carcinoma during Nucleos(t)ide Analogue Therapies: A Multicenter Retrospective Study

Jun Inoue, Tomoo Kobayashi, Takehiro Akahane, Osamu Kimura, Kosuke Sato, Masashi Ninomiya, Tomoaki Iwata, Satoshi Takai, Norihiro Kisara, Toshihiro Sato, Futoshi Nagasaki, Masahito Miura, Takuya Nakamura, Teruyuki Umetsu, Akitoshi Sano, Mio Tsuruoka, Masazumi Onuki, Hirofumi Niitsuma, Atsushi Masamune and THERME Study Group †

† THERME Study Group includes the following members: Yutaka Mano (Sendai Medical Center), Noriatsu Kanno (Abies Kanno Clinic), Shu Abe (JR Sendai Hospital), Atsushi Kanno (Sendai Red Cross Hospital), Takayuki Kogure (Tohoku Medical Pharmaceutical University Hospital), Takehiko Igarashi (Osaki City Hospital), Osamu Kido (Iwate Prefectural Chuo Hospital) and Noriyuki Obara (Japan Community Health Care Organization Sendai Hospital).

Supporting information

Figure S1. Response rates for NA therapies based on ALT normalization and HBV DNA undetectability at 1 to 5 years of therapy. Three types of criteria for ALT normalization were as follows. WHO criteria: male, ≤30; female, ≤19. AASLD criteria: male, ≤35; female, ≤25. JSH criteria: ≤30.

Figure S2. Cumulative incidences of HCC according to the achievement of ALT normalization 2 years after the start of NA therapy that were evaluated with different criteria and those according to the viral response. (**a-b**) Comparison of cumulative incidences of HCC between patients who achieved ALT normalization based on the criteria of WHO (**a**) and AASLD (**b**). In these analyses, patients who developed HCC before 24 months and those whose observation periods were less than 24 months were excluded. (**c-d**) Comparison of cumulative incidences of HCC between patients with and without detectable HBV DNA at 1 year (**c**) and 2 years (**d**) of therapy.

Figure S3. Analysis of patients whose ALT was abnormal (>30) at 1 year of therapy but was normalized at 2 years. (**a**) Numbers of patients according to the status of ALT normalization at 1 and 2 years. (**b**) Among only patients without ALT normalization at 1 year of therapy, a comparison of cumulative HCC incidences

between patients with and without ALT normalization at 2 years was performed.

Table S1. Comparison of baseline characteristics between patients with ALT normalization (JSH criteria, ≤30) and those without after propensity score matching at 1 year of NA therapies.

**Parameter**	**ALT ≤ 30 at 1 year (n = 119)**	**ALT >30 at 1 year (n = 119)**	***p*-Value**
Age (years)	50 (41-58)	50 (40-61)	0.788
Sex (male/female)	90/29	92/27	0.760
Previous IFN (+/-)	14/105	12/107	0.678
DM (+/-)	9/110	6/113	0.424
T-Bil (mg/dl)	0.8 (0.6-1.1)	0.8 (0.6-1.2)	0.347
AST (U/l)	42 (29-82)	51 (35-75)	0.084
ALT (U/l)	56 (34-128)	60 (44-109)	0.295
Alb (g/dl)	4.2 (3.9-4.5)	4.2 (3.9-4.5)	0.879
Cr (mg/dl)	0.77 (0.67-0.88)	0.78 (0.69-0.87)	0.775
PLT ($\times 10^4/\mu l$)	17.6 (13.6-22.4)	16.2 (13.2-21.9)	0.477
AFP (ng/ml)	4.2 (3.0-9.5)	5.6 (3.1-11.4)	0.385
HBV DNA (log IU/ml)	5.3 (4.3-6.7)	5.4 (4.8-6.5)	0.451
HBsAg (IU/ml)	886 (151-2614)	1247 (216-3770)	0.377
HBeAg (+/-)	38/67	40/64	0.734
HBcrAg (log U/ml)	4.4 (3.0-6.4)	3.5 (3.0-6.1)	0.718
FIB-4 index	1.61 (1.08-2.83)	2.10 (1.21-3.00)	0.302
HBV genotype (A/B/C/D)	2/35/60/0	1/32/77/2	0.232
Initial NA (LAM/ETV/TDF/TAF)	16/89/9/5	12/88/13/6	0.705
Observation period (months)	72 (45-110)	72 (44-108)	0.817

Fig. S1

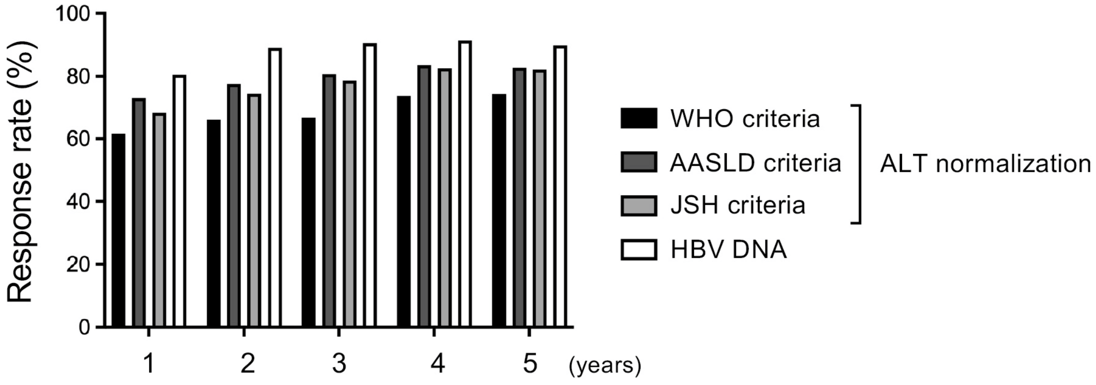
Response rate (%)
100
80
60
40
20
0
1
2
3
4
5
(years)
WHO criteria
AASLD criteria
JSH criteria
HBV DNA
ALT normalization

Fig. S2

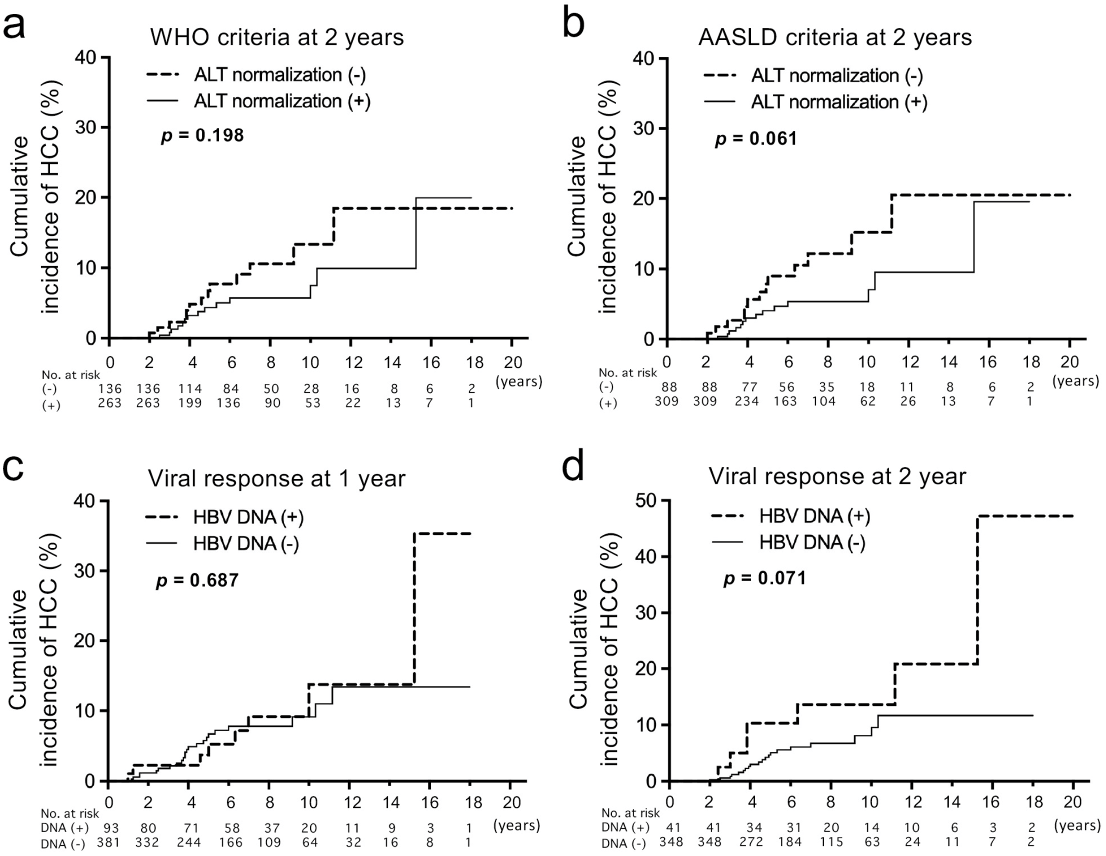
a
WHO criteria at 2 years
ALT normalization (-)
ALT normalization (+)
p = 0.198
Cumulative incidence of HCC (%)
No. at risk
(-) 136 136 114 84 50 28 16 8 6 2
(+) 263 263 199 136 90 53 22 13 7 1
(years)
b
AASLD criteria at 2 years
ALT normalization (-)
ALT normalization (+)
p = 0.061
Cumulative incidence of HCC (%)
No. at risk
(-) 88 88 77 56 35 18 11 8 6 2
(+) 309 309 234 163 104 62 26 13 7 1
(years)
c
Viral response at 1 year
HBV DNA (+)
HBV DNA (-)
p = 0.687
Cumulative incidence of HCC (%)
No. at risk
DNA (+) 93 80 71 58 37 20 11 9 3 1
DNA (-) 381 332 244 166 109 64 32 16 8 1
(years)
d
Viral response at 2 year
HBV DNA (+)
HBV DNA (-)
p = 0.071
Cumulative incidence of HCC (%)
No. at risk
DNA (+) 41 41 34 31 20 14 10 6 3 2
DNA (-) 348 348 272 184 115 63 24 11 7 2
(years)

Fig. S3

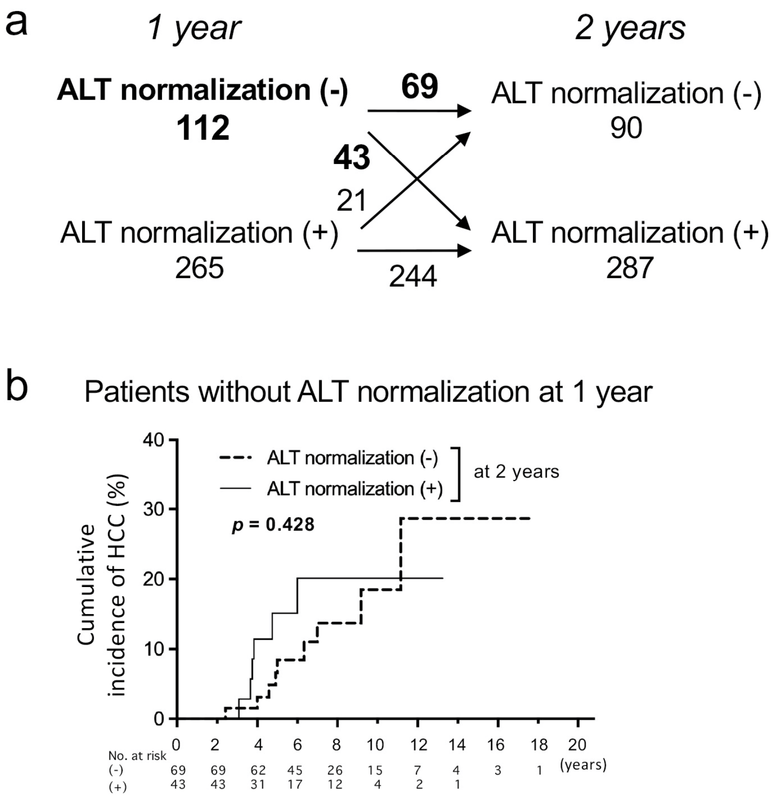

a
1 year
2 years
ALT normalization (-)
112
69
ALT normalization (-)
90
43
21
ALT normalization (+)
265
244
ALT normalization (+)
287
b
Patients without ALT normalization at 1 year
Cumulative incidence of HCC (%)
ALT normalization (-)
ALT normalization (+)
at 2 years
p = 0.428
0
10
20
30
40
0 2 4 6 8 10 12 14 16 18 20 (years)
No. at risk
(-) 69 69 62 45 26 15 7 4 3 1
(+) 43 43 31 17 12 4 2 1
